# Bone Marrow Gene Therapy for HIV/AIDS

**DOI:** 10.3390/v7072804

**Published:** 2015-07-17

**Authors:** Elena Herrera-Carrillo, Ben Berkhout

**Affiliations:** Laboratory of Experimental Virology, Department of Medical Microbiology, Center for Infection and Immunity Amsterdam (CINIMA), Academic Medical Center, University of Amsterdam, Amsterdam 1105 AZ, The Netherlands; E-Mail: e.herreracarrillo@amc.uva.nl

**Keywords:** bone marrow, hematopoietic stem cell (HSC), virus, antiviral, gene therapy, lentiviral vector, HIV-1, RNAi

## Abstract

Bone marrow gene therapy remains an attractive option for treating chronic immunological diseases, including acquired immunodeficiency syndrome (AIDS) caused by human immunodeficiency virus (HIV). This technology combines the differentiation and expansion capacity of hematopoietic stem cells (HSCs) with long-term expression of therapeutic transgenes using integrating vectors. In this review we summarize the potential of bone marrow gene therapy for the treatment of HIV/AIDS. A broad range of antiviral strategies are discussed, with a particular focus on RNA-based therapies. The idea is to develop a durable gene therapy that lasts the life span of the infected individual, thus contrasting with daily drug regimens to suppress the virus. Different approaches have been proposed to target either the virus or cellular genes encoding co-factors that support virus replication. Some of these therapies have been tested in clinical trials, providing proof of principle that gene therapy is a safe option for treating HIV/AIDS. In this review several topics are discussed, ranging from the selection of the antiviral molecule and the viral target to the optimal vector system for gene delivery and the setup of appropriate preclinical test systems. The molecular mechanisms used to formulate a cure for HIV infection are described, including the latest antiviral strategies and their therapeutic applications. Finally, a potent combination of anti-HIV genes based on our own research program is described.

## 1. Introduction

The HIV epidemic, first recognized in 1981, remains one of the major threats to human health. Globally, approximately 35 million people are living with HIV and more than 25 million people have died of HIV-related causes [1]. Finding a safe, effective, and durable HIV vaccine remains a top priority, but despite more than 30 years of research there is still no vaccine that provides effective protection against HIV infection [2,3]. Combination antiretroviral therapy (cART) can significantly prolong the life of HIV-infected individuals. Some studies found no evidence for HIV-1 evolution in patients on suppressive cART [4], but other reports suggested that cART does not fully suppress viral replication and does not eliminate the viral reservoirs [5,6]. Moreover, the toxicity associated with the life-long adherence to cART, together with the appearance of drug-resistant HIV variants in some patients, supports the continuous search for novel drug and original approaches to fight HIV [7]. Gene therapy has emerged as a promising approach for the treatment of HIV/AIDS as it may facilitate the sustained inhibition of HIV replication after a single therapeutic intervention. A single proof of concept was provided by the so-called “Berlin patient,” who remained free of detectable HIV after receiving a bone marrow transplant from a CCR5-Δ32 homozygous donor [8,9]. This genetic defect could be mimicked in a gene therapy setting.

**Figure 1:**
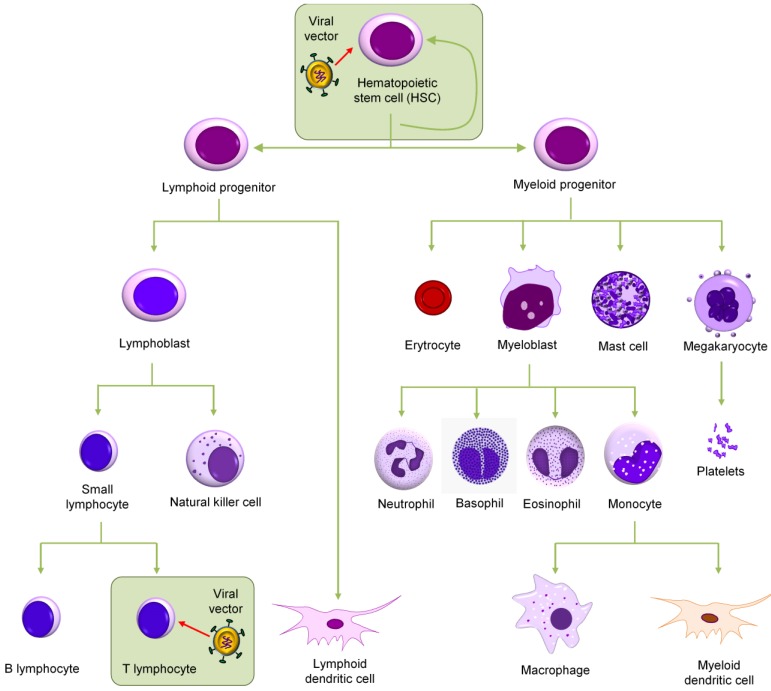
Target cells for an anti-HIV gene therapy. Shown is the scheme of hematopoiesis. Either hematopoietic stem cells (HSCs) from bone marrow or the mature CD4^+^ T cells can be targeted. These two cell populations are boxed.

HIV mainly targets CD4^+^ T cells by binding to the CD4 molecule as well as a chemokine co-receptor, usually CCR5 or CXCR4, on the cell surface. In addition macrophages, monocytes, and dendritic cells can be infected by HIV. In theory, both peripheral blood T cells and HSCs from the bone marrow can be selected as the target cells for an anti-HIV gene therapy (Figure 1). However, because T cells have a limited life span and because HIV also infects other cell types of the hematopoietic lineage, it is thought to be a significant advantage to transduce the HSC precursors. The most important characteristics of HSC are their capacity for self-renewal and their ability to restore all blood cell lineages after bone marrow ablation. HSC will differentiate into diverse hematopoietic lineages, supplying the immune system with HIV-resistant cell types that subsequently colonize the blood and tissues. The classic source of HSCs is bone marrow. For more than 45 years, physicians punctured the marrow and drew out the bone marrow cells with a syringe. In the early 1990s, the human umbilical cord and placenta were recognized as a rich source of HSCs. On the other hand, it was known for decades that a small number of HSCs circulate in blood (1 log less than their counterpart in the marrow). In the past 10 years, researchers have developed a safe way to efficiently mobilize HSCs from the marrow to the blood by pre-treating the donor with granulocyte-colony stimulating factor (G-CSF). There are no indications of qualitative differences in the differentiated cells derived from peripheral blood, cord blood, and bone marrow. Therefore, the most common method for HSC isolation nowadays is apheresis of peripheral blood because the harvest of cells is easier, with minimal discomfort for the donor, e.g., no requirement for anesthesia and a hospital stay. In this review we discuss the biological perspective for transducing HSCs with an anti-HIV gene and the many options available for choosing the therapeutic gene. We also discuss the gene transfer vector of choice and its design. Finally, we propose a combination of anti-HIV genes to increase the genetic threshold for viral escape based on our own research line.

## 2. Selection of Therapeutic Targets

Two gene-based approaches for immune reconstitution of HIV-infected individuals have been developed to date. One approach is based on “artificial T cell receptors.” Although patients naturally develop CD8^+^ T cell responses (cytotoxic T lymphocyte) during the acute phase of HIV-1 infection, this does not suffice for blocking virus replication and does not result in clearance of the virus. The establishment of a latent HIV-1 reservoir forms a major obstacle to viral clearance in the chronic phase of infection. A strategy to clear virus from the body may be to enhance the recognition of HIV-1 infected cells by engineering T cells. The second approach is based on “intracellular immunization” that aims to protect cells from infection based on the intracellular expression of antiviral genes [10]. The gene products used to combat HIV infection can be classified into two groups: protein and RNA-based inhibitors. We will first discuss T cell engineering.

### 2.1. Artificial T Cell Receptors

Initial studies performed in the early 1990s described the potential for enhancing the recognition of HIV-1 infected cells by expressing a molecularly cloned T cell receptor (TCR) specific to HIV proteins, termed artificial TCR. Preclinical *in vivo* studies demonstrated suppression of HIV in humanized mice that were injected with T cells expressing the artificial Gag-SL9 TCR [11]. However, effective HIV-specific cytotoxic T lymphocytes are restricted by HLA class I alleles and therefore cannot be applied universally. To avoid HLA restriction of artificial TCRs, T cells can be engineered to express a chimeric antigen receptor (CAR), which combines the specificity of an antibody and the intracellular signaling capacity of a T cell receptor. Preclinical studies of CD8^+^ T cells engineered to express CAR have demonstrated antigen-specific proliferation, inhibition of HIV replication, and cytolytic activity against HIV-infected T cells [12,13]. A recent study based on the results of three clinical trials (clinical trial NCT01013415 and [12,14]) indicated that CAR gene therapy is safe [15]. However, the level of CAR-modified cells was found to decrease over time, thus limiting the durability of the anti-HIV effect [12], but other studies reported a remarkable persistence of CAR-modified cells [14,15,16]. Engineering of HSCs to express CAR molecules may allow for the prolonged production of long-lived non-susceptible cells and eliminate the risk of generating self-reactive hybrid TCR pairs because modified cells would be naturally selected in the thymus [17].

### 2.2. Intracellular Immunization

**Figure 2:**
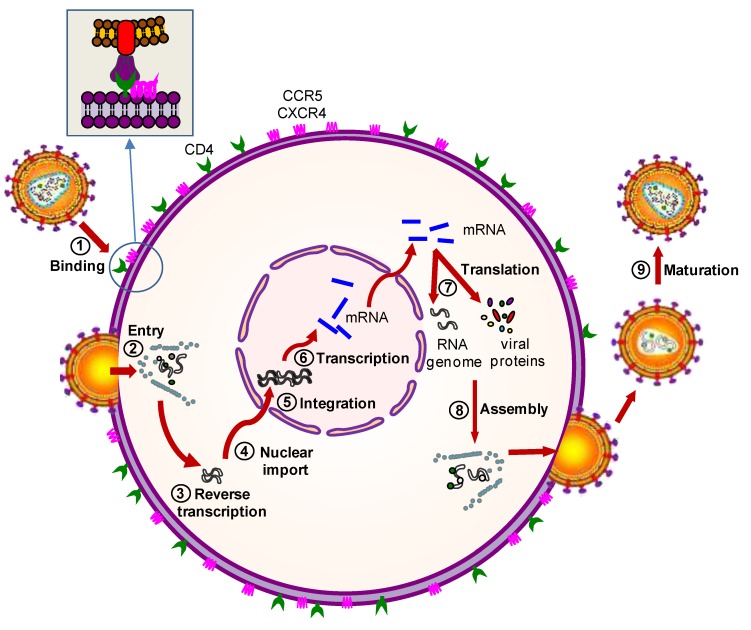
Steps of the HIV-1 replication cycle that can be targeted by gene therapy. The HIV-1 replication steps that can be targeted by gene therapy are shown: (**1**) HIV-1 binding to cell membrane; (**2**) HIV-1 entry into the cell; (**3**) reverse transcription; (**4**) transport of the HIV-1 proviral genome into the nucleus; (**5**) integration of the viral genome into the cellular DNA; (**6**) transcription of the HIV-1 proviral genome; (**7**) translation of the viral messenger RNA (mRNA) into new viral proteins; (**8**) virion assembly inside the cell; and (**9**) maturation of the immature virion into a completely infectious particle.

Over the last two decades several anti-HIV gene therapy approaches have been developed. The anti-HIV gene products will interfere with crucial steps of the viral replication cycle or target a cellular factor that is required for virus replication. Figure 2 illustrates the steps of HIV-1 replication cycle that can be targeted. The viral replication cycle is arbitrarily divided into two stages: the early stage refers to the steps of infection from cell binding to the integration of the viral DNA into the cell genome, whereas the late stage begins with viral gene expression from the integrated provirus and leads to the release of the immature virions that subsequently mature into infectious particles (for further details, see the Figure legend). Initially, anti-HIV genes were designed to inhibit HIV transcription or translation, which occur during the late stage of the replication cycle. More recently, laboratories have developed anti-HIV therapies based on the inhibition of early replication stage, which may be beneficial for a robust antiviral effect. According to the gene products used to combat HIV infection, the gene therapy strategies are classified into two groups: protein and RNA-based therapies.

#### 2.2.1. Protein-Based Therapies

Proteins can be engineered to inhibit either viral or cellular targets. Protein-based strategies include trans-dominant negative proteins (an altered form of a viral or cellular protein that can inhibit the normal function of its wild-type counterpart), fusion inhibitors (a protein or peptide that affects the fusion process during viral entry into the cell), intrabodies (recombinant antibodies expressed intracellularly), intrakines (modified intracellular chemokines), host restriction factors, and nucleases. The first protein tested in a HIV gene therapy trial was an altered form of the HIV Rev protein termed RevM10. RevM10 is a trans-dominant Rev mutant that interferes with the normal Rev function and thereby prevents the export of unspliced genomic HIV RNA from the nucleus to the cytoplasm [18,19,20]. Cells expressing RevM10 were shown to have a survival advantage in HIV-infected individuals, but no substantial impact on the viral load was observed [21,22]. Other trans-dominant negative HIV-1 proteins have been developed, including Tat mutants that prevent transactivation of the viral LTR promoter [23] and Vif- and Gag-based trans-dominant inhibitors [24,25,26,27]. Mutants of cellular proteins that act as co-factors of virus replication have also been evaluated as trans-dominant negative molecules (e.g., S6 [28], Sam68 [29,30], or HDAC1 [31]).

A different protein-based approach is based on secreted antiviral proteins. Several laboratories have genetically engineered truncated and soluble forms of CD4 (sCD4) that exhibit antiviral properties [32,33,34]. However, the secretion level of sCD4 was too low to efficiently inhibit HIV. Recently, promising results were obtained with a fusion inhibitor based on a 46 amino acid domain of the HIV-1 Envelope gp41 protein (C46) that prevents membrane fusion [35]. The C46 peptide has also been stably expressed as a membrane-anchored peptide (maC46) that was able to inhibit replication of a broad range of HIV-1 isolates [36,37]. The safety of maC46 has been confirmed in a phase I clinical trial in which autologous T cells, transduced with a retroviral vector expressing maC46, were infused into patients [38]. However, the *in vivo* antiviral effect remains currently unknown. Secreted neutralizing antibodies or intrabodies have been developed against the viral Tat, Vif, Reverse Transcriptase, and Integrase proteins and have been shown to inhibit virus replication in gene-modified cells *in vitro* [39,40,41,42,43,44]. However, the neutralization breadth of these intrabodies was limited. For instance, although the anti-Tat intrabody was expected to increase the relative survival of transduced cells *in vivo* by blocking the Tat-TAR axis that controls viral gene expression, the number of transduced cell level progressively decreased and was too low to cause a therapeutic effect [44]. In another attempt to inhibit HIV entry, several modified intracellular chemokines or intrakines were designed to block the surface expression of HIV-1 CCR5 co-receptor, but modified cells retained a residual co-receptor level on the cell membrane that suffices for HIV-1 entry [45,46,47].

An alternative strategy to block the expression of HIV-1 co-receptors is to engineer nucleases that are specific for sequence motifs in the co-receptor genes. Various approaches have been explored to enable selective gene editing: zinc finger nucleases (ZFNs), transcription activator-like effector nucleases (TALENs), and, most recently, CRISP/Cas9 nucleases. Most studies have focused on the genes encoding the HIV-1 co-receptor CCR5 and CXCR4, but the integrated HIV-1 DNA genome can also be targeted [48,49,50,51,52,53,54,55,56]. The major hurdle with genome-editing systems concerns the possible off-target effects that may lead to non-specific genome modifications. This issue must be solved before genome editing can be considered for anti-HIV-1 gene therapy. Recently, several groups demonstrated that antiviral restriction factors can also be exploited for anti-HIV gene therapy applications [57]. For instance, the human TRIMcyp protein was shown to potently inhibit HIV in human T cells and macrophages *in vitro* and *in vivo* [58]. Nevertheless, the introduction or overexpression of exogenous restriction factors may induce an unwanted immune response that eventually results in the removal of the modified cells. Thus, the feasibility of this approach needs to be addressed in larger clinical trials.

We also would like to mention some exciting recent studies on gene therapy vectors that should provide protection against HIV-1 transmission. This novel strategy is called Vectored Immuno Prophylaxis or VIP and is based on the production of an anti-HIV transgene-encoded antibody. VIP yields immunological protection (like a vaccine), but without actively invoking the immune system (antigen design, production and vaccination) [59,60,61]. In particular, VIP yielded sustained expression of multiple antibodies at a high level upon a single intramuscular injection in mice [62]. These antibodies confer anti-HIV activity and protected humanized mice against an HIV-1 challenge [62,63].

#### 2.2.2. RNA-Based Therapies

Similar to anti-HIV proteins, RNAs can be designed to target either viral or cellular products. RNA molecules have an advantage over proteins because nucleic acids are not immunogenic and RNA-based therapies may therefore be more suitable for long-term applications. In addition, RNA-based therapies mostly work in a sequence-specific manner, thus avoiding adverse effects in the cell. Although cellular targets are less prone to mutations that may trigger viral escape, there may be serious side effects of downregulating cellular products. Therefore, HIV-1 products have been the preferred target for antiviral RNAs. RNA-based strategies include antisense, ribozymes, aptamers, small interfering RNAs (siRNAs), short hairpin RNAs (shRNAs), and the related AgoshRNA design that was recently discovered. The agents that act through the RNA interference (RNAi) mechanism: siRNA, shRNA, and AgoshRNA will be explained in more detail because they form a more recent addition to the antiviral arsenal.

To date numerous antisense RNA molecules have been designed to target HIV-1 mRNAs in a sequence-specific manner, resulting in the formation of non-functional RNA duplexes that are subsequently destroyed. Antisense RNA molecules against the HIV-1 trans-activation response element (TAR) and Envelope-encoding sequences of HIV-1 RNA have been designed and shown to inhibit HIV *in vitro* [64,65,66]. Ribozymes are similar to antisense RNA molecules but have an added catalytic activity. Ribozymes have been directed against tat/vpr [67,68,69], rev/tat [70], and U5 leader sequences [71] of the HIV-1 RNA genome and have shown promising antiviral activity *in vitro* [72,73]. However, the ribozyme-only treatments have not demonstrated a therapeutic effect in clinical trials [69]. RNA aptamers do not attack the HIV RNA genome. Instead, these oligonucleotides competitively bind and sequester specific molecular targets, thus inhibiting their biological function. Anti-HIV-1 aptamers are mainly based on TAR and Rev-responsive elements (RRE), thus neutralizing the action of the HIV-1 proteins Tat and Rev, respectively. The inhibitory effect of TAR and RRE aptamers was reported *in vitro* [74,75,76,77] and *in vivo* [78,79], but no antiviral effect was observed in the only clinical trial to date that exclusively used aptamers [80].

**Figure 3:**
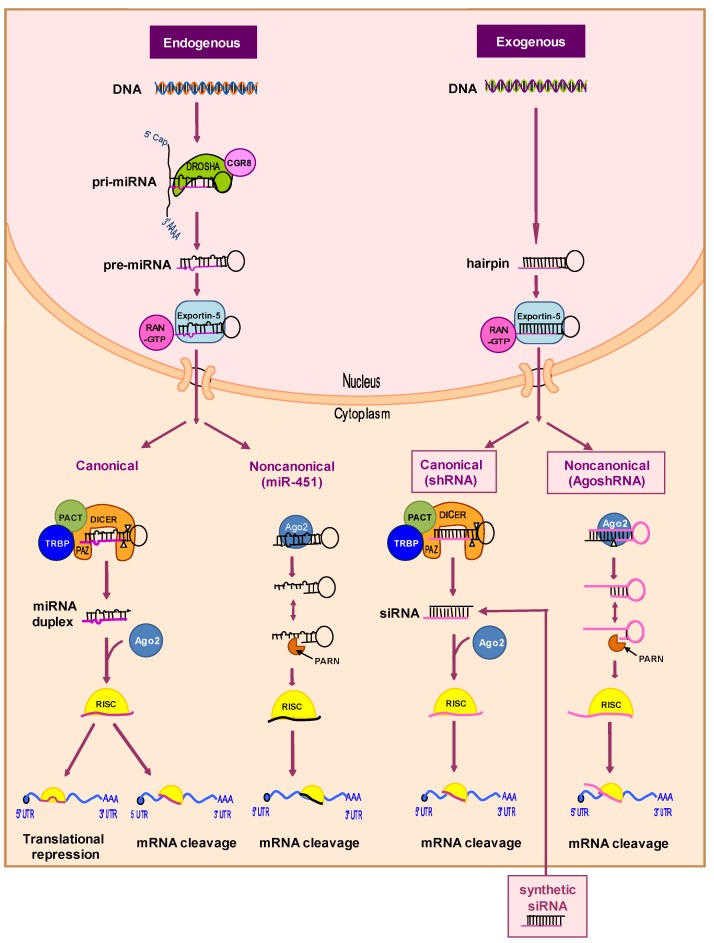
The endogenous miRNA and exogenous shRNA processing pathways. The intracellular processing pathways are depicted starting from the miRNA gene of the cell (endogenous) or the transduced shRNA gene cassette (exogenous). The canonical Dicer-dependent and noncanonical Dicer-independent pathways are depicted for both molecules. Ago2 plays an essential role in Dicer-independent pathways. See the text for further details. PACT: Protein activator of protein kinase R; Pri-miRNA: Primary miRNA; Ago2: Argonaute 2 nuclease; RISC: RNA-induced silencing complex; TRBP: Transactivation response RNA-binding protein.

More recently, RNAi has evolved as a powerful tool to regulate gene expression post-transcriptionally in a sequence-specific manner. The RNAi mechanism uses double-stranded RNA molecules (dsRNA) to trigger mRNA degradation (Figure 3). First, a primary miRNA transcript (pri-miRNA) is made, of which a hairpin-like RNA structure is processed by the “Microprocessor” complex, which consists of the Drosha nuclease and its dsRNA-binding partner DGCR8. The resulting pre-miRNA is cleaved near the terminal loop by the Dicer nuclease in collaboration with the trans-activation response RNA-binding protein (TRBP) and protein activator of PKR (PACT) cofactors [81]. This miRNA pathway yields the mature RNA duplex, of which one strand of approximately 22 nucleotides is preferentially loaded into the Argonaute (Ago) enzyme as part of the RNA-induced silencing complex (RISC). The miRNA-loaded RISC complex targets a partially complementary mRNA transcript for degradation and/or translational repression. This mechanism is conserved in all eukaryotes and can be exploited for therapeutic gene silencing. The canonical RNAi pathway can be triggered by artificial substrates as synthetic siRNAs that can be transfected into the cells (Figure 3, right, canonical pathway) [82]. Antiviral activity of siRNA molecules targeting the HIV RNA genome (Nef, Tat, Gag, Vif, Env) or the mRNA for important cellular co-factors has been reported in short-term virus replication experiments [83,84,85,86,87,88]. However, HIV-1 causes a chronic infection and infected patients require long-term treatment, which cannot easily be accomplished for synthetic siRNAs, in part due to inefficient delivery of nucleic acids into cells.

An alternative to the regular injection of exogenous siRNA is the expression of shRNAs from a transgene construct in the target cells [89,90]. The shRNA is synthesized in the nucleus, transported to the cytoplasm by the Exportin-5 protein, and processed by the Dicer endonuclease into siRNAs of ~21 bp with 2-nt 3′ overhangs (Figure 3, right, canonical pathway) [91]. The “passenger” strand of the siRNA is degraded and the “guide” strand programs RISC to cleave the perfectly complementary target mRNA. Recent evidence indicates that one can also design Dicer-independent shRNAs [92]. We recently identified a specific shRNA design with a short stem and small loop that triggers this alternative processing route [93,94]. This special shRNA design was named AgoshRNA to reflect the dual role of Ago2 in processing and subsequently target RNA cleavage. The AgoshRNA design possess several potential advantages over regular shRNAs, but more studies are required to demonstrate their knockdown efficacy [93].

AgoshRNAs may become the silencing method of choice in diverse situations, e.g., in cells such as monocytes that lack a significant amount of Dicer [95]. In addition, saturation of Dicer as a critical component of the cellular RNAi pathway may not occur with AgoshRNAs. Moreover, only a single RNAi-active guide strand is produced, which is an important feature to restrict RNAi-induced off-target effects due to the passenger strand. Ago2-mediated processing of shRNAs also yields more precise ends compared to Dicer processing, which is notoriously inaccurate [96,97]. Finally, AgoshRNAs may mimic the Dicer-independent cellular miR-451 that is loaded exclusively into Ago2, thus avoiding off-target effects via Ago1, 3, and 4 [98]. However, it remains to be seen whether the regular shRNA or novel AgoshRNA design yields more effective molecules and we think that the latter design can still be improved [99,100].

## 3. Combinatorial Approaches

HIV mutates rapidly due to the high viral turn-over in patients and the significant mutation frequency of the HIV-1 Reverse Transcriptase enzyme, which lacks a proofreading mechanism. Thus targeting of a host cell co-factor may represent a less escape-prone antiviral option. However, host targeting may cause cytotoxicity and viral escape would be possible through adaptation to an alternative cellular co-factor. Like the use of a single antiviral drug, gene therapy with a single anti-HIV gene is prone to virus escape. Therefore, effective gene therapy applications against HIV will likely require a combination of anti-HIV genes targeting different HIV-1 components or important co-factors [90,101,102]. Besides additive inhibition, this combinatorial approach will raise the genetic threshold for the evolution of drug-resistant virus variants as multiple mutational hits will be required in multiple target sites. Accordingly, several groups have incorporated multiple anti-HIV genes into a single vector.

**Figure 4:**
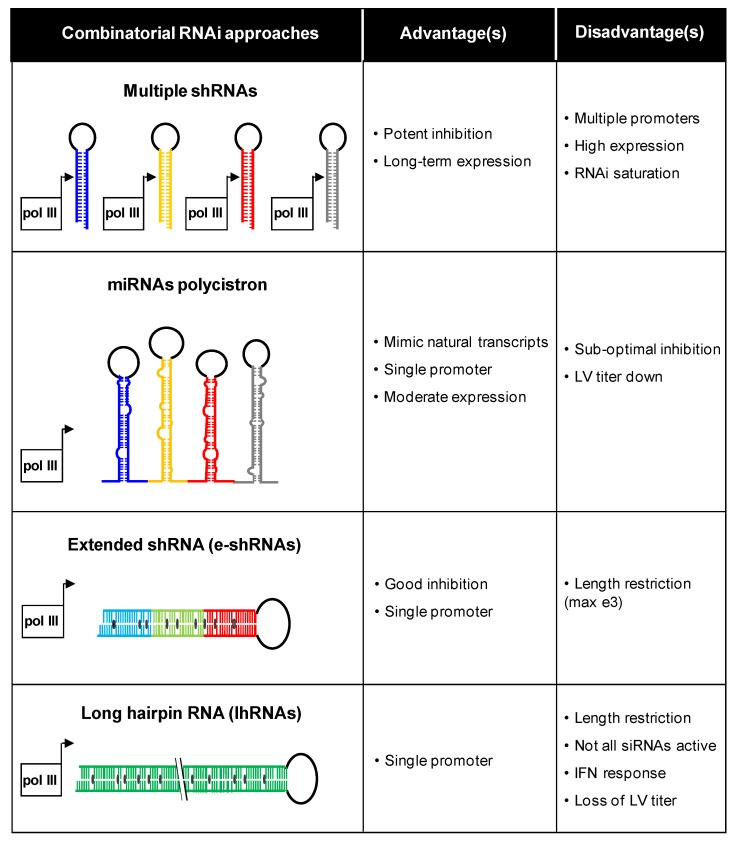
Combinatorial RNAi strategies. Four inhibitory scenarios are plotted with the respective advantages and disadvantages. This figure was adapted from [111]. LV: lentiviral vector.

Our group has investigated different combinatorial RNAi approaches (Figure 4). For instance, multiple shRNA cassettes can be combined in the same vector [103]. Different promoters were used to express three shRNAs against HIV-1 to avoid recombination-mediated deletion of shRNA cassettes on repeated promoter sequences. Multiple inhibitors can also be generated from a polycistronic miRNA transcript [104,105]. A recent study showed that multiple shRNAs can be effectively expressed from a single vector via tandem repeats of different miRNA-based backbones [106]. This strategy is called multiplexed miRNA-based shRNAs (shRNA-miRs). When peripheral blood mononuclear cells from HIV-1 seropositive individuals were transduced with a shRNA-miRs vector and transplanted into mice, efficient suppression of virus replication and restoration of the CD4 T cell count was observed. Alternatively, extended shRNAs (e-shRNA) expressing two or three siRNAs or long hairpin RNAs (lhRNA) encoding many siRNAs can be designed [107,108,109,110]. However, most silencing activity is lost for the extended RNA duplex designs. Figure 4 lists the major advantages and disadvantages of the different combinatorial RNAi strategies [111].

The RNAi inhibitors can be combined with other protein and RNA-based inhibitors [102] or conventional antiretroviral drugs [112,113]. For instance, one study employed a combination of CCR5 shRNA, chimeric human/rhesus TRIM5a, and a TAR aptamer and a different study employed a TAR aptamer, CCR5 ribozyme, and a tat-rev shRNA [114,115]. These studies demonstrated no apparent toxicity for the combination of antiviral genes. In a separate study, a combination of the membrane-anchored peptide (maC46) and multiple tat/rev shRNAs were combined in a single vector [116]. Our group demonstrated that “second-generation” shRNAs can also be combined with protease inhibitors to avoid the evolution of clinically relevant drug-resistance mutations in the protease-encoding gene [113]. One could even create hybrid molecules that combine siRNA and other antiviral activities, e.g., an RNA aptamer that binds to and neutralizes the viral Envelope protein [117,118]. Although these results are promising, the key question is whether these compounds can effectively block viral escape.

## 4. Vector Choice

To fight chronic diseases like HIV infection, stable transduction of target cells with the transgene is desirable to avoid repeated administration of anti-HIV molecules. To achieve a durable therapeutic effect, viral vectors that integrate into the host genomes seem the preferred route. Mostly vectors derived from gamma-retroviruses (murine leukemia virus) and lentiviruses (HIV-1) have been used in clinical trials to treat HIV-1 infection [38,102]. The viral genome has been truncated and modified in a number of ways to generate vectors that are safe for clinical use. Both gamma-retroviral and lentiviral vectors (LV) integrate randomly into the host cell genome [119]. As a consequence, integration of the vector may cause insertional oncogenesis. Gamma-retroviruses usually integrate near transcriptional start sites, and severe side effects and the induction of leukemia have been reported in clinical trials [120]. LVs tend to integrate within introns of transcribed regions, thereby limiting their potential to cause insertional oncogenesis [121]. Nevertheless, one should remain careful because some clonal expansion of HIV-infected cells has been observed in infected patients [122,123]. Nevertheless, the development of leukemia due to an HIV-1 integrating event has never been observed in HIV-1 infected individuals. An additional advantage of the LV design is that this vector can infect dividing cells as well as non-dividing cells and terminally differentiated cells, while the gamma-retroviral vector can only infect dividing cells. Given the lower risk of insertional oncogenesis and the ability to effectively transduce many cell types, including HSC, we have chosen the LV system for delivery of the antiviral payload.

**Figure 5:**
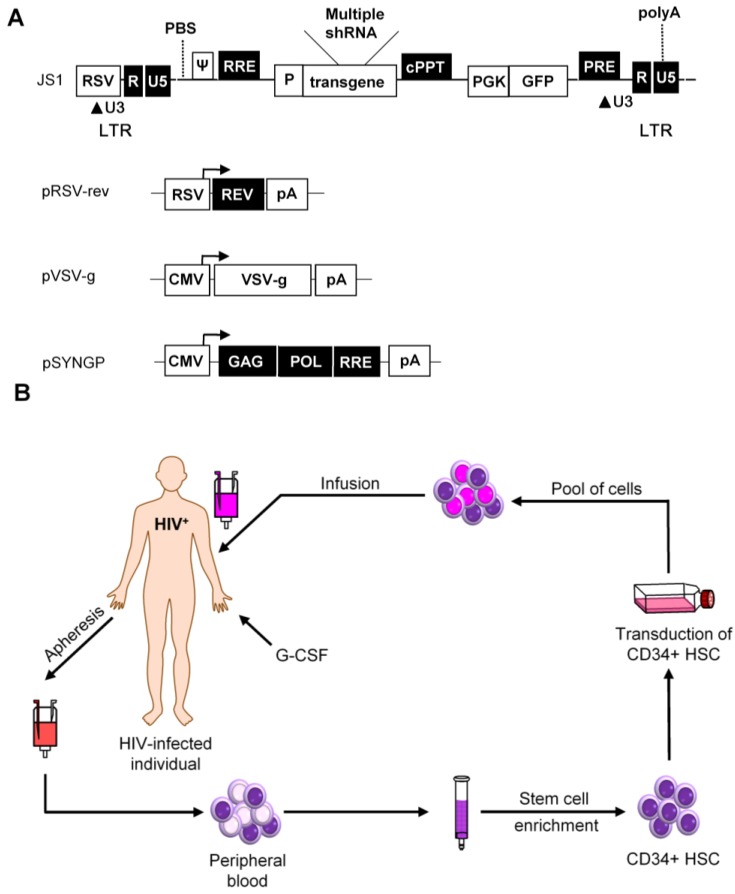
Self-inactivating lentiviral vectors for stable shRNA expression. (**A**) The lentiviral vector JS1 is shown with three plasmids needed for lentiviral vector production. The vector genome is expressed from the Rous Sarcoma Virus (RSV) promoter. Transcripts start with the HIV-1 R and U5 regions and the packaging signal (ψ). The enhanced green fluorescent protein (GFP) reporter is expressed from the phosphoglycerate kinase promoter (PGK). Transcription of the vector genome and the GFP reporter terminates at the HIV-1 polyA signal within the 3′ LTR; (**B**) Scheme of a hematopoietic stem cell (HSC) clinical trial. An HIV-infected patient who fails on regular drug therapy will undergo apheresis for the collection of CD34^+^ HSC after pretreatment with granulocyte-colony stimulatory factor (G-CSF). The mixed cell population containing CD34^+^ HSC will be purified and transduced *ex vivo* with the therapeutic construct. Transduced cells will be infused back into the patient and the antiviral gene should protect these cells against HIV-1.

The first demonstration of HSC transduction with a LV was presented in 1996 and since then this vector has been extensively modified to increase the efficiency and safety [124]. The HIV genome encodes nine genes, of which four are dispensable for *in vitro* virus propagation [125]. Therefore, these four accessory genes were deleted, together with the wild-type envelope gene (Figure 5A). An envelope protein of a different virus is included instead, most often the glycoprotein of vesicular stomatitis virus (VSV) due to its broad target cell tropism [124]. In addition, Gag-Pol (SYNGP), Rev (RSV-rev), and VSV-g genes are separated on plasmids and expressed from a heterologous promoter, while the region in the viral vector between the Long Terminal Repeats (LTRs) is replaced with the therapeutic gene on a fourth plasmid to minimize the risk of generating a replication-competent virus through recombination. Further improvement of the biosafety was achieved by construction of self-inactivating (SIN) vectors. SIN vectors contain a deletion in the U3 region of the 3′ LTR that is transferred to the 5′ LTR promoter during reverse transcription, leading to transcriptional inactivation of the vector in transduced cells [126,127]. This self-inactivating vector design diminishes the risk of oncogene activation by promoter insertion and reduces the risk of vector mobilization and recombination with the wild-type virus [128].

Another biosafety-related issue is the administration of a minimal amount of viral particles to prevent side effects. To allow the use of a minimal dose of viral particles for cellular transduction, the third generation LV has an optimized transduction efficiency using a central polypurine tract (cPPT) sequence and a post-transcriptional regulatory element (PRE) from the woodchuck hepatitis virus. For the development of a clinical gene therapy application, it is essential that the vector can be produced to high titers and that the vector is genetically stable. LV is efficient in transducing CD4^+^ T cells and HSCs. However, the LV system is based on HIV-1, which may complicate its use as a vehicle to deliver anti-HIV-1 genes, e.g., RNAi inducers. We previously discussed these potential problems and presented protocols to effectively use LV for an RNAi-based attack on HIV-1 [129,130,131]. Briefly, it is important to avoid targeting of HIV-1 sequences that are also present in the LV system. This requirement can frequently be met by using codon-optimized versions of the gag/pol and rev vectors that were generated to improve safety (marked in black in Figure 5A). Another potential issue is that the anti-HIV gene may target sequences in the LV, thus reducing the production or titer by “self-targeting.” This problem can be avoided by careful selection of shRNAs that do not target parts of the LV backbone.

To date, the LV system has been shown to be effective for *in vitro* delivery, integration, and stable expression of transgenes in the hematopoietic system. For a durable HIV-1 treatment, one can transduce HSC *ex vivo* with a LV encoding different HIV specific inhibitors (Figure 5B). The transduced cells should constitutively express the inhibitors and thus be resistant to HIV-1 infection. The protected HSC are engrafted back into the patient, where they will give rise to an HIV-1 resistant myeloid and lymphoid cell population. This approach would ideally only need a single infusion. Engraftment of autologous transduced HSC will not only result in a steady production of HIV-1 resistant T cells, but also other HIV-1 susceptible cells, e.g., macrophages and monocytes. Gene-modified cells will theoretically have a survival advantage over non-modified cells in HIV-infected individuals as the infected cells will be recognized and destroyed by the patients’ immune system. Thus, the autologous transplant should eventually be able to (partially) reconstitute the immune system.

## 5. Preclinical Safety and Efficacy Tests

For a discussion of the required preclinical safety and efficacy tests, we would like to focus on the research line that we have developed since 2004. We proposed an RNAi-based gene therapy for the durable control of HIV-1 infection. We started by screening many shRNA candidates that target important HIV-1 sequences and selected the most potent inhibitors. These shRNAs target highly conserved viral sequences, with 100% sequence identity among at least 70% of HIV-1 genomes of all subtypes. We could make a human T cell line fully resistant to HIV-1 by expression of a single shRNA, but we also described viral escape by the selection of a point mutation in the target sequence [87,90,132,133]. We therefore designed LV that express a combination of four shRNAs (R4A) that target highly conserved sequences in the HIV-1 RNA genome. The safety and efficacy of the combinatorial RNAi-based gene therapy can first be probed *in vitro*. We tested the four candidate shRNAs in a competitive cell growth (CCG) assay based on the difference in proliferation rate of shRNA-transduced (GFP-positive) and untransduced cells in the same culture. We observed a negative impact of one of the four shRNAs on *in vitro* cell growth [134]. The safety of this combinatorial RNAi approach should also be tested in appropriate *in vivo* models to prepare for a clinical trial in humans. In particular, we used the BRG-HIS mouse model in which immunodeficient newborn mice are injected with human hematopoietic progenitor cells. These mice build a fairly complete human immune system consisting of different cell lineages, including mature T cells. This complex process of hematopoiesis can be monitored to screen for a negative impact of the HSC gene therapy on cell development. For instance, we tested the impact of LV-transduced anti-HIV shRNAs cells and observed normal development of the human immune system and no adverse effects for three of the four shRNAs tested. The shRNA that showed some adverse effects *in vivo* also demonstrated a negative effect in the CCG assay, which allowed us to reformulate the shRNA cocktail [135]. The three nontoxic and potent antiviral shRNAs were used to design the combinatorial RNAi vector R3A. Each shRNAs targets a highly conserved sequence of the HIV-1 RNA genome encoding the Integrase, Protease and Tat-Rev proteins [90]. Other therapeutics approaches (e.g., zinc-finger nucleases or ZFN) were also tested in this pre-clinical mouse model [136].

The efficacy of the R3A vector was shown *in vitro* in experimental settings with pure cultures of modified T cells and subsequently with mixed cultures of modified and unmodified cells [135,137]. It was confirmed that no viral escape occurs with the combinatorial R3A regimen in prolonged mixed cultures, which renders this combination therapy durably effective. It is also important to test the impact of HIV genetic diversity on the combinatorial RNAi regimen proposed. Therefore, the efficacy of R3A was tested against a broad panel of HIV isolates and subtypes. The results demonstrate the broad effectiveness of the triple shRNA regimen [138]. Based on these promising *in vitro* results, the therapeutic potential of this combinatorial RNAi approach should be tested in appropriate *in vivo* models. These studies are currently being addressed in the BRG-HIS mouse model.

## 6. Clinical Studies

We consider a lentiviral gene therapy for HIV-infected patients who fail on regular ART, where autologous HSCs will be transduced *ex vivo* with the LV encoding the triple shRNAs. In theory, the transduced HSCs will durably supply all derived immune cells with the combination of antivirals. Because preferential survival of the shRNA-expressing cells over unprotected cells in the presence of HIV-1 is expected, perhaps a single gene therapy treatment will suffice. Theoretically, unprotected cells will be infected and removed by the immune system. This survival benefit of protected cells should result in a gradual increase in the percentage of cells that are not susceptible to HIV-1 infection, restoration of the damaged immune system, and hopefully a blockage of disease progression towards AIDS. Although the feasibility of such a cell-based gene therapy has recently been demonstrated in a pilot clinical study [139], successful application of modified HSCs for HIV treatment requires further optimization to improve the transduction efficiency and enhance the engraftment of the modified cell population. In this regard, the use of mild myeloablation methods may facilitate more efficient engraftment, an approach that can be used in a safe manner in a gene therapy setting [140,141].

To date, only a few anti-HIV gene therapy protocols have progressed towards clinical trials, mostly addressing the feasibility and safety of gene-transduced autologous HSC transplantation in patients. Table 1 summarizes some of the clinical trials performed with gene-modified HSC to date. We did not list the trials that target mature T cells.

**Table 1:** Clinical trial of HIV gene therapy based on modified HSC transplantation.

Gene Therapy Mechanism	Phase	Reference(s)
Rev-responsive element decoy (Rev protein)	Pilot	[80]
Trans-dominant Rev (Rev protein)	I–II	[142]
Ribozyme (Tat/Rev mRNA)	II	[69,143]
		NCT00074997
		NCT00002221
	II	NCT01177059
Combinatorial trans-dominant Rev (Rev protein) and antisense (Pol mRNA)	I–II	NCT00003942
Combinatorial strategy: fusion inhibitor C46 (Env protein) and shRNA (CCR5)	I–II	NCT01734850
Combinatorial strategy: shRNA (Tat/Rev mRNA), TAR decoy (Tat protein) and ribozyme (CCR5)	Pilot	[102] NCT00569985
		NCT01153646

## 7. Conclusions

Although current cART can potently reduce the plasma HIV viral load to undetectable levels in most patients, it is not curative. cART remains expensive because of the need for life-long drug adherence. Other cART problems are the side effects observed in some patients after long-term drug intake and the possibility that drug-resistant virus variants evolve. Although HSC gene therapy will also be expensive, it presents an attractive alternative that has the potential to control HIV infection with a single treatment. Genetically modified HSCs and all derived cell types will continuously produce the anti-HIV genes. Anti-HIV genes can be designed to interfere with crucial steps of the viral replication cycle either by targeting a viral factor or a cellular factor required for virus replication. For the success of a HSC gene therapy, it is pivotal that therapeutic genes potently and permanently inhibit viral replication. The anti-HIV genes should exhibit long-term efficacy without exerting adverse effects on lineage specific differentiation and cellular functions, and appropriate gene delivery vehicles with minimal toxicity are thus essential. There is growing evidence that the third generation lentiviral vector, which allows stable integration of the therapeutic gene, can be used safely [144,145,146]. Although the feasibility of HSC gene therapy has been demonstrated recently in a pilot clinical study [139], successful application of modified HSCs for HIV treatment requires further optimization, e.g., by improving the transduction efficiency and enhancing the engraftment of the modified cell population. Given the recent technological advances in the field, we believe that HSC modification forms an attractive means to develop a durable therapy for HIV infection. HSC gene therapy may also be combined with approaches that specifically target the HIV-1 reservoirs to achieve at least a “functional cure” (the virus is still present, but suppressed in the absence of antiretroviral drugs), since a “complete cure” (virus eradication from an infected individual) seems like an impossible mission at the moment.
